# When Ear Pain Signals More: Otomastoiditis-Related Bilateral Cranial Neuropathies in an Immunocompromised Patient

**DOI:** 10.7759/cureus.107605

**Published:** 2026-04-23

**Authors:** Shrutikona Das, Sarah M Yi, Yoo Mee Shin

**Affiliations:** 1 Department of Medicine, Emory University School of Medicine, Atlanta, USA; 2 Hospital Medicine, Emory University Hospital Midtown, Atlanta, USA

**Keywords:** complications of acute mastoiditis, cranial neuropathy, diabetes mellitus type 2, fungal infection, immunocompromised patients

## Abstract

Otomastoiditis is the infection and inflammation of both the otitis media (middle ear) and the mastoid air cells. Involvement of the mastoid air cells can occur if the infection extends past the middle ear to affect contiguous structures. Fungal mastoiditis is very rare and almost exclusively seen in immunocompromised patients. We present an unusual case of a type 2 diabetic with fungal otomastoiditis manifesting as left-sided ear pain, as well as neuropathies of the left cranial nerve VII and right cranial nerve III. While facial nerve paralysis is often attributed to idiopathic causes, it is important that physicians maintain a high index of suspicion, especially when multiple cranial nerves are involved.

## Introduction

Mastoiditis is a rare disease, especially in the adult population, with an incidence of less than 2 per 100,000 population [1]. *Streptococcus pneumoniae*, *Streptococcus pyogenes*, *Staphylococcus aureus*, and *Haemophilus influenzae* are the most common bacterial pathogens implicated in mastoiditis, which are unsurprisingly the same pathogens most implicated in acute otitis media [2,3]. The most common fungal pathogens include *Candida* and *Aspergillus* species, with *Aspergillus niger*, *Aspergillus fumigatus*, and *Aspergillus flavus* being the species most frequently reported [4,5].

Management of otomastoiditis typically involves antimicrobial treatment directed at the causative pathogen, myringotomy, and incision and drainage of the periosteal space when indicated [6]. Complications include tympanic membrane perforation, abscess formation, labyrinthitis, sinus thrombosis, cranial nerve (CN) VI or CN VII palsies, and skull base osteomyelitis [4,7]. We present an unusual case of fungal otomastoiditis with skull base involvement resulting in bilateral cranial neuropathies.

This article was previously presented as a meeting abstract at the 2025 Southern Hospital Medicine Conference on September 25, 2025, the 2025 ACP Georgia Annual Scientific Meeting on October 24, 2025, and the 2026 SGIM Southern Regional Meeting on February 28, 2026.

## Case presentation

A 74-year-old patient with newly diagnosed chronic lymphocytic leukemia (CLL), type 2 diabetes mellitus (T2DM) complicated by peripheral neuropathy, chronic kidney disease stage 3a, and peripheral vascular disease presented with left-sided otalgia, left-sided CN VII palsy, and right-sided CN III palsy. He had been on meropenem for several months for chronic *Pseudomonas* spp. osteomyelitis of his right foot.

Three weeks before admission, the patient presented to his primary care provider with left ear pain and a sore throat. At this time, he was also noted to have right-sided ptosis and diplopia on upward gaze. One week later, he was evaluated by an otolaryngologist and diagnosed with otitis externa with concern for fungal infection. He underwent two washouts of the external auditory canal. Several days later, he developed left-sided facial paralysis, prompting him to seek emergency care. Unfortunately, records of these encounters were unavailable for our review.

On admission, examination of his left ear revealed thick debris with granulation tissue at the inferior tympanic membrane. Neurological examination demonstrated intermittent binocular diplopia on horizontal and vertical extremes with spared pupillary function. He had right-sided ptosis, and his right eye rested in a downward and externally deviated position with restricted upward and medial movement. Facial sensation was intact. He achieved a grade V on the House-Brackmann scale for left-sided facial paralysis, with incomplete eye closure, restricted forehead motion, and minimal mouth movement. Table 1 highlights a basic differential based on clinical presentation.

**Table 1:** Differential diagnoses of bilateral cranial nerve palsies.

Diagnosis	Common clinical presentation	Typical diagnostic study findings
Guillain-Barré syndrome	Progressive ascending paralysis, sensory deficits, and cranial nerve deficits	Albuminocytologic dissociation on cerebrospinal fluid (CSF)
Demyelinating disease (e.g., multiple sclerosis)	Varies by location of the lesion	MRI showing demyelination in the brain and/or spinal cord
Myasthenia gravis	Fluctuating muscle weakness and ocular symptoms such as diplopia and ptosis	Serology showing acetylcholine receptor-binding antibodies or muscle-specific kinase antibodies
Meningitis	Headache, fever, neck stiffness, altered mental status, seizures, aphasia, hemiparesis, coma, and focal neurologic deficits, including cranial nerve palsies	*Bacterial*: CSF cell counts with neutrophil-predominant pleocytosis, low glucose, and positive CSF culture and/or DNA polymerase chain reaction (PCR). *Viral*: lymphocyte-predominant pleocytosis on CSF PCR. *Fungal*: lymphocyte-predominant pleocytosis, PCR, and fungal serological markers such as CSF galactomannan and β-D-glucan
Brainstem mass	Cranial nerve palsies, ataxia, paresthesia, headache, and paresis	MRI showing a brainstem mass
Brainstem stroke	Acute onset of vertigo, hemiplegia, ataxia, altered mental status, dysarthria, or dysphagia	MRI brain showing an infarct

**Figure 1:**
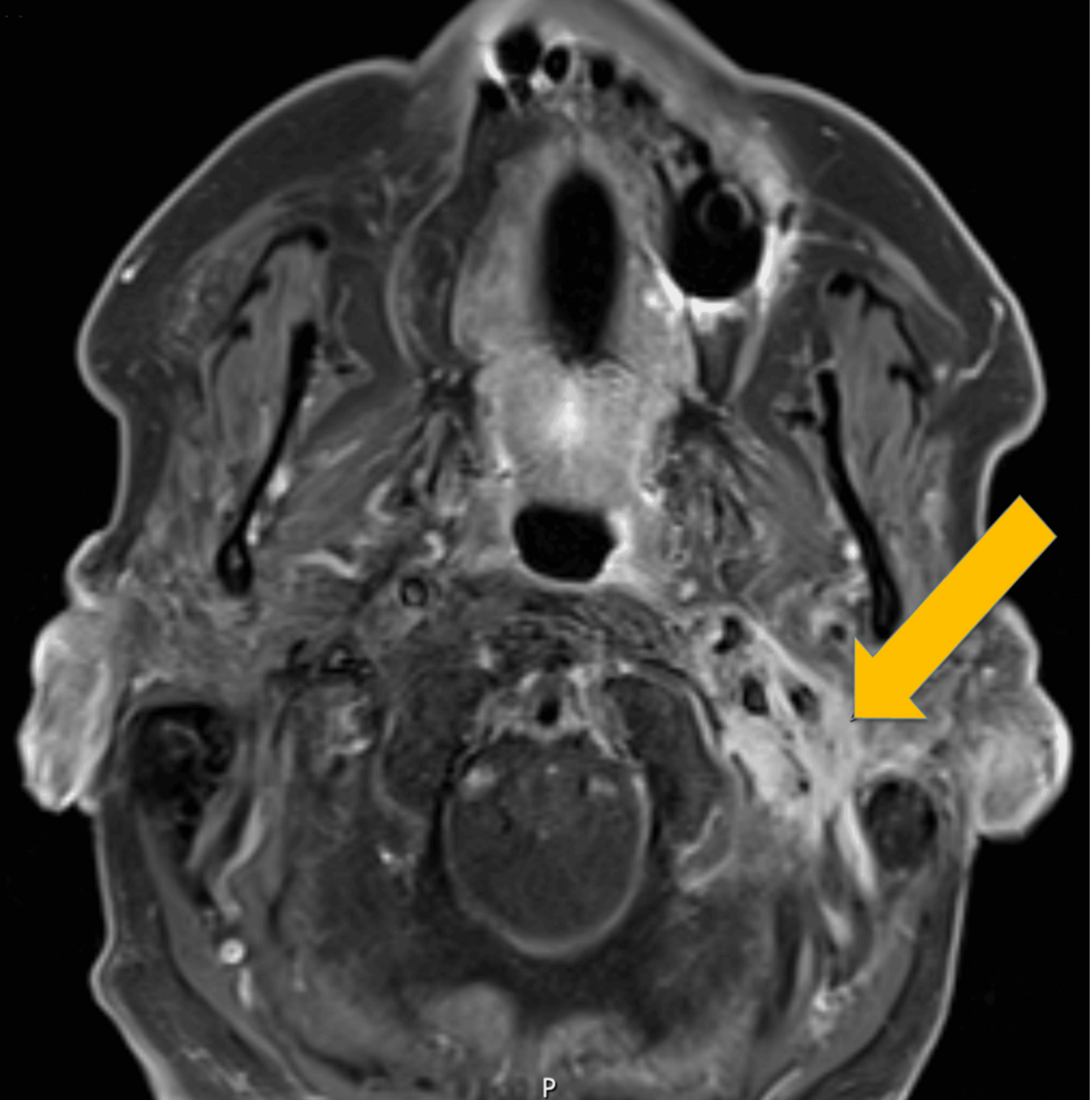
MRI of the brain, axial section, T1, fat saturation with contrast, showing complete opacification of left mastoid air cells with erosions of the mastoid septae and enhancing debris throughout the left mastoid cavity extending into the left middle ear.

Imaging included CT of the internal auditory canals and posterior fossa with IV contrast, as well as MR of the brain, face, and orbits with and without IV contrast. The left temporal bone demonstrated complete opacification of mastoid air cells, enhancing edematous changes extending into the stylomastoid region, and dehiscence of the sigmoid plate and tegmen mastoideum with focal pachymeningeal enhancement along the floor of the middle cranial fossa. Additionally, enhancing inflammatory changes predominantly involving the tympanic and mastoid segments of the left facial nerve were observed, compatible with facial neuritis secondary to infection. Although no drainable pyogenic abscess was identified, small collections of purulent material were suspected within the left mastoid and adjacent soft tissues inferior to the skull base (Figure 1).

**Figure 2:**
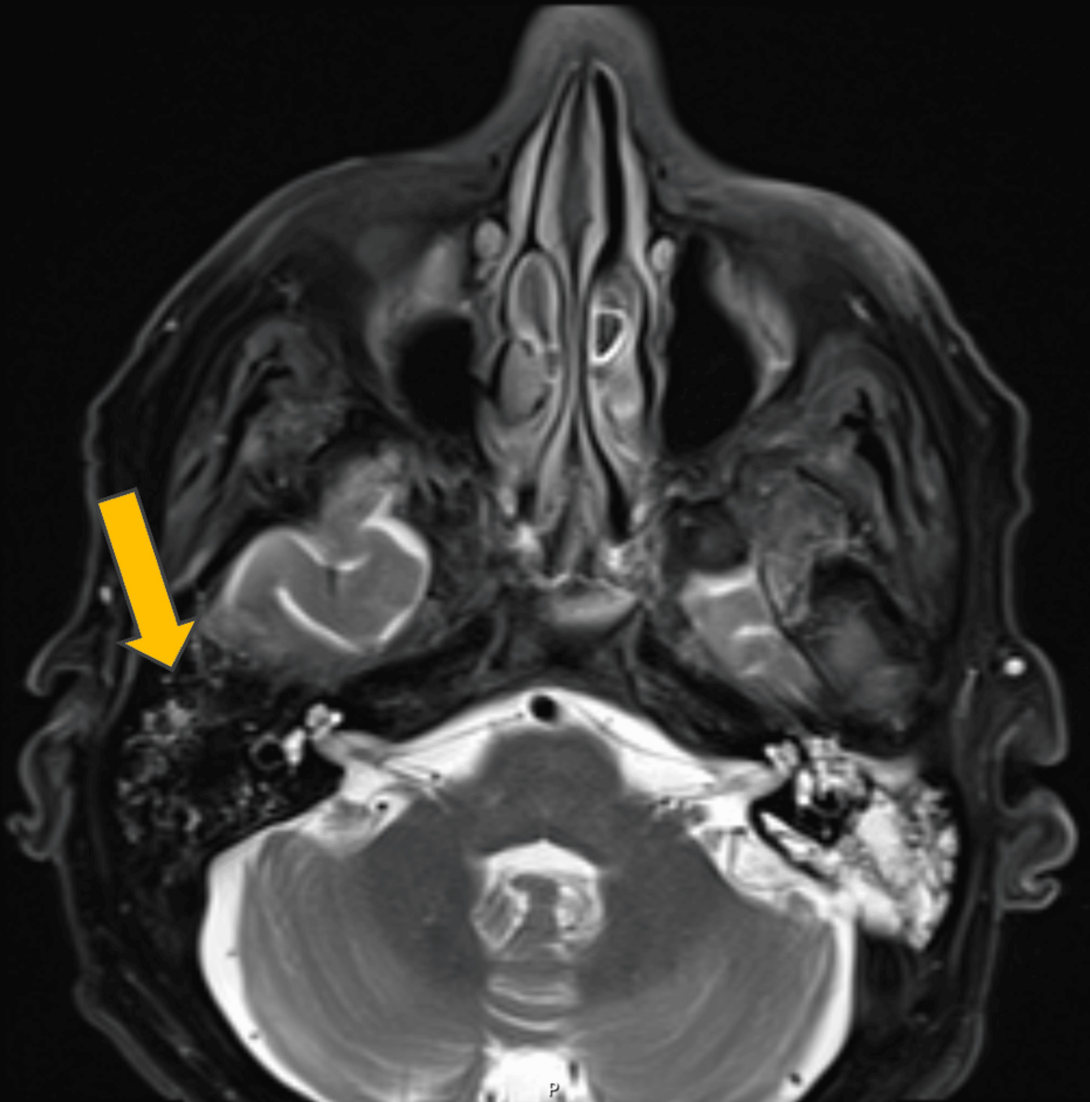
MRI of the brain, axial section, T2, fat saturation, showing small right mastoid effusion in addition to the opacification of left mastoid air cells.

On the right, no enhancement of CN III was observed; however, a small mastoid effusion and enlargement of the cavernous sinus were noted. No filling defect was present, although thrombosis or infection remained possible given the clinical context (Figure 2). The orbits had an expected appearance bilaterally.

Labs were notable for negative blood cultures, mild leukocytosis of 9.3 K/µL, hemoglobin of 7.9 g/dL, and A1c of 7.7%. Acetylcholine receptor binding antibodies were negative. Serum galactomannan was negative, whereas β-D-glucan was positive. Though tissue culture of debris in the external auditory canal grew both *S. epidermidis* and *A. flavus/oryzae*, tissue biopsy of granulation tissue in the ear canal showed only gram-positive cocci. Mastoid bone biopsy was deferred. Cerebrospinal fluid (CSF) analysis revealed scant lymphocytic pleocytosis but was otherwise negative. No growth was noted on CSF cultures.

A 10-day course of acyclovir monotherapy was initiated for left-sided CN VII paralysis due to prior adverse effects to corticosteroids. He was also treated with linezolid, which was transitioned to doxycycline after two weeks, along with isavuconazole to minimize nephrotoxicity.

One month after discharge, the patient’s contralateral right-sided CN III palsy and left otalgia resolved. At two months, repeat MRI demonstrated significant radiographic improvement of the inflammation, though he continued to experience tenderness in the left mastoid area and persistent CN VII palsy. Planned facial reanimation surgery was deferred after he noted gradual clinical improvement after four months. Doxycycline and isavuconazole were discontinued after five months, and he continues to be monitored without further antimicrobial therapy.

## Discussion

Fungal mastoiditis is rare and almost exclusively seen in immunocompromised hosts. Cranial nerve involvement, particularly CN VII, is uncommon but well-documented. In a retrospective study of 109 patients with complicated acute otitis media, 16.7% developed unilateral facial nerve palsy [7]. However, bilateral cranial neuropathies are exceedingly rare, with only limited cases reported in the literature. Prior reports include bilateral facial nerve palsies in a pediatric patient with mastoiditis [8], and in two adults with otitis media associated with myeloperoxidase antineutrophil cytoplasmic antibody [9]. Per our literature review, this case represents one of the few reported cases of fungal otomastoiditis presenting with noncontiguous bilateral cranial neuropathies.

In this case, a patient with T2DM developed coalescent otomastoiditis with skull base involvement and possible right-sided cavernous sinus thrombosis. The left-sided CN VII palsy was likely secondary to invasive aspergillosis. In contrast, the right-sided CN III palsy likely had a multifactorial etiology, including diabetic microvascular ischemia, cavernous sinus syndrome secondary to infection or possible thrombosis, or extension of the infection from the skull base. The rapid improvement of the right-sided CN III palsy with antimicrobial therapy suggests an infectious rather than a purely microvascular etiology.

Skull base involvement is a recognized complication of otomastoiditis, and typically occurs through direct extension from the mastoid air cells to the adjacent bony structures, though hematogenous and soft tissue spread may also occur [10]. The pathway of CN VII and CN III elucidates the proximity of these structures in the setting of infectious spread. CN VII is supplied by four nuclei found in the pons, medulla, and upper cervical spinal cord. It passes into the internal acoustic meatus and temporal bone close to the inner and middle ear, before exiting the skull via the stylomastoid foramen. It then enters the parotid gland, where it diverges into five branches across the facial region [11]. CN III originates from two nuclei in the midbrain and exits the brainstem near the midline. It enters the lateral aspect of the cavernous sinus and proceeds into the supraorbital fissure before finally reaching the orbit [12].

Although this patient was diagnosed with CLL, his immunoglobulin levels were within normal limits, and he was under surveillance, given his mild disease. He lacked other risk factors for invasive fungal infection aside from T2DM. While invasive fungal infections are uncommon in immunocompetent hosts, a case review of *Aspergillus* otomycosis showed how poorly controlled diabetes is associated with immune dysregulation that increases susceptibility to opportunistic infections, where five patients had T2DM as their only underlying risk factor [13].

Patients with diabetes are more susceptible to infection due to the induction of senescence and impaired immune cell proliferation [14]. The combination of aging and hyperglycemia promotes a state of chronic low-grade inflammation, which cascades into the overproduction of reactive oxygen species and pro-inflammatory cytokines. These processes contribute to pancreatic β-cell destruction, insulin deficiency, immune cell senescence in the bone marrow, and reduced lymphopoiesis [14]. Chronic hyperglycemia has also been shown to impair multiple immune response mechanisms, including the suppression of cytokine production (tumor necrosis factor-alpha, interleukin (IL)-1, and IL-6), leukocyte recruitment, pathogen recognition, immune cell dysfunction, and complement activation [15]. These changes impair both innate and adaptive immunity, resulting in higher infection rates and reduced responses to vaccines [14]. Though this patient’s A1c was only moderately elevated at 7.7%, the chronicity of his disease likely contributed to impaired immunity, making him more susceptible to infection.

Despite discordant fungal serological results, *Aspergillus* spp. were likely the primary driver for this patient’s otomastoiditis. When evaluated separately, the β-D-glucan assay has higher sensitivity for invasive aspergillosis (81%) than the galactomannan assay (49%) at the cost of lower specificity (82% versus 97%) [16]. A meta-analysis of 13 studies found that concordant positive galactomannan and β-D-glucan tests incur nearly 100% specificity but relatively low sensitivity (49%) [17]. Defining suspected invasive aspergillosis based on seropositivity of either assay increases the sensitivity to 89%, with only a modest reduction in specificity to 86% [16].

Both tissue culture and histopathology have limited sensitivity in diagnosing invasive aspergillosis. Many patients with positive serological markers have negative cultures, with one study revealing that 25% to 50% of hematopoietic cell transplant patients with invasive disease had negative cultures [18]. Although histopathology helps establish a definitive diagnosis, it is only slightly more sensitive than tissue culture [19].

In this case, *Aspergillus* spp. were identified on culture but not on biopsy. However, this does not preclude invasive aspergillosis given the known limitations of tissue sampling and the positive β-D-glucan assay. It is unlikely that *S. epidermidis* was the primary pathogen responsible for this patient’s coalescent otomastoiditis, as it is considered a contaminant in many instances of otitis media [20]. Given the clinical presentation, imaging findings, and microbiological data, invasive fungal otomastoiditis was the most likely diagnosis. Nonetheless, he was treated with both linezolid/doxycycline and isavuconazole to cover both gram-positive and fungal organisms and responded adequately to therapy.

For a diabetic patient presenting with atypical or multiple cranial neuropathies, even in the absence of classic immunocompromise, maintaining a broad differential is integral to ensuring timely diagnosis and treatment, as it can significantly improve patient outcomes.

## Conclusions

Although most cases of facial nerve paralysis are idiopathic, thorough investigation for alternative causes is essential for appropriate management, especially when multiple cranial nerves are affected. This case highlights the potential for T2DM-associated immune dysregulation to facilitate invasive otomastoiditis with skull base involvement and noncontiguous bilateral cranial neuropathies. Despite prolonged broad-spectrum antibiotic therapy, immunocompromised patients remain susceptible to infections from organisms not covered by the antibiotic. Early recognition, extended antimicrobial therapy, and close longitudinal follow-up are critical to achieving better health outcomes.
